# Distribution and Antimicrobial Resistance of *Salmonella* Isolated from Pigs with Diarrhea in China

**DOI:** 10.3390/microorganisms6040117

**Published:** 2018-11-26

**Authors:** Jin-Hui Su, Yao-Hong Zhu, Tian-Yi Ren, Liang Guo, Gui-Yan Yang, Lian-Guo Jiao, Jiu-Feng Wang

**Affiliations:** College of Veterinary Medicine, China Agricultural University, Beijing 100193, China; sujh0209@gmail.com (J.-H.S.); zhu_yaohong@hotmail.com (Y.-H.Z.); day2seven@sina.com (T.-Y.R.); binghaitiankong20@163.com (L.G.); yangguiyan1990@hotmail.com (G.-Y.Y.); jiaolianguo@sina.com (L.-G.J.)

**Keywords:** *Salmonella*, pig, antimicrobial resistance, multilocus sequence typing, monophasic

## Abstract

*Salmonella* can cause enteric diseases in humans and a wide range of animals, and even outbreaks of foodborne illness. The aim of this study was to investigate the frequency and distribution of serovars, and antimicrobial resistance in *Salmonella* isolates from pigs with diarrhea in 26 provinces in China from 2014 to 2016. A total of 104 *Salmonella* isolates were identified and the dominant serovar was *S.* 4,[5],12:i:- (53.9%). All *Salmonella* isolates were resistant to trimethoprim-sulfamethoxazole, and many were resistant to ampicillin (80.8%) and tetracycline (76.9%). Among 104 *Salmonella* isolates, *aac(6′)-Ib-cr* was the dominant plasmid-mediated quinolone resistance gene (80.8%), followed by *qnrS* (47.1%). The pulsed-field gel electrophoresis results suggest that the *Salmonella* isolates from different regions were genetically diverse, and ST34 was the most prevalent. *S.* 4,[5],12:i:- isolates is the widespread presence of heavy metal tolerance genes. The fact that the same sequence types were found in different regions and the high similarity coefficient of *S.* 4,[5],12:i:- isolates from different regions indicate the clonal expansion of the isolates, and the isolates carried various antimicrobial resistance genes. The multidrug resistant *Salmonella* can be widely detected in pigs, which will present a challenge for farm husbandry.

## 1. Introduction

*Salmonella* is Gram-negative pathogenic intestinal species of bacteria of the Enterobacteriaceae family. The World Health Organization (WHO) has estimated that *Salmonella* is responsible for 550 million infections among humans each year. In the USA population, approximately 1.2 million infections are caused by *Salmonella* and the economic loss owing to salmonellosis is estimated to be $3.5 billion annually [1]. Despite the lack of official surveillance data on *Salmonella* in China, it is estimated that it causes 75% of foodborne diseases [2].

More than 2500 serovars have been identified, and these serovars are classified based on slide agglutination with O and H antigen-specific sera. Some serovars are host specific, but others can infect a variety of hosts. Pigs are considered to be one of the major environmental reservoirs of *Salmonella* [3]. 

*Salmonella*, especially *S. enterica*, is one of the most common pathogens to cause diarrhea in pigs. *S. enterica* can infect pigs of all ages, especially weaned pigs and young growing-finishing pigs [4]. These pigs can be susceptible to salmonellosis as a result of their immature immune systems, the existence of concurrent diseases, and the stress that they undergo, including the stress due to weaning, transportation, and experiencing new environments. The rate of *S. enterica* infection among weaned and finishing pigs is high, and salmonellosis among pigs can cause reduced weight gain and even death [5]. Therefore, it is critical for the swine industry to implement strategies to reduce horizontal transmission and infection with *Salmonella*.

Antimicrobials are important to prevent and treat bacterial infections. However, due to their misuse, drug resistance is increasing and multidrug-resistant strains have developed.

Fluoroquinolone are the drugs of choice to treat complicated cases of life-threatening salmonellosis [6]. The emergence of fluoroquinolone resistant *Salmonella* strains in several serovars poses a serious threat to public health, and the resistance rate to fluoroquinolone of *Salmonella* isolates has increased dramatically around the world [7]. The mutations of plasmid-mediated quinolone resistance (PMQR) and quinolone resistance-determining regions (QRDRs) mechanism contribute to the resistance of quinolone and fluoroquinolone antimicrobials [8].

Colistin is being intensively used as a last-resort antimicrobial drug for treating infections with carbapenemase-producing *Enterobacteriaceae* [9]. In veterinary medicine, colistin mainly to treat Gram-negative infections of the intestinal tract, and used as a growth promoter in some countries [10]. In 2015, the emergence of a plasmid-mediated colistin resistance mechanism involving the *mcr-1* gene was firstly reported in *Escherichia coli*, and caught world-wide attention [9].

*Salmonella* 4,[5],12:i:-, a monophasic variant of *Salmonella* Typhimurium that lacks the second-phase flagellar antigen, was first reported in the 1980s [11]. There has seen a rapid worldwide emergence of *S.* 4,[5],12:i:- in the past two decades. *S.* 4,[5],12:i:- is one of the most common serovars isolated from foodstuffs and animals in Europe, and now ranks as the top serovar among both human and veterinary isolates in some European countries [12]. There are two major clonal lines was identified, “Spanish clone” and “European clone” [13]. *S.* 4,[5],12:i:- is strongly associated with pig and pork products [14].

China has the largest number of live pigs in the world (http://www.fao.org/faostat/en/#data). However, there has been no specific information on the *Salmonella* strains in pigs in China. In spite of many studies have been showed epidemiological and phenotypic features of *S.* 4,[5],12:i:-, little is known about the ecological success of *S.* 4,[5],12:i:- circulating in humans and animals in China. We investigated the frequency and distribution of *Salmonella* in pigs isolated from 2014 to 2016 in various provinces in China. We also compared the antimicrobial resistance phenotypes, pathogenicity, heavy metal tolerance and genetic diversity between *S.* Typhimurium and *S.* 4,[5],12:i:- to investigate the possible factors that involved in the widespread of *S.* 4,[5],12:i:- isolates.

## 2. Materials and Methods

### 2.1. Sample Collection

A total of 492 samples were obtained from 66 farms in 26 provinces. These samples were derived from the intestinal contents of pigs that died of diarrhea or from the feces of pigs with diarrhea. The 26 provinces were selected randomly from the 34 provinces in China, and the 66 farms in these provinces were selected by convenience sampling (i.e., they were the farms that were the easiest to request samples from). The 492 pigs comprised all the pigs with diarrhea on the selected farms. All the samples were kept at 4 °C during transport and storage before analysis.

### 2.2. Isolation of Strains 

For each sample, 10 g was added to 100 mL of buffered peptone water (Luqiao, Beijing, China), homogenized using a stomacher, and incubated at 37 °C for 16–20 h to produce pre-enriched broth. Subsequently, 300 µL of the pre-enriched broth was transferred into Modified Semi-Solid Rappaport-Vassiliadis (MSRV) medium that was modified by adding novobiocin (A) (Luqiao, Beijing, China). The inoculated media were incubated at 42 °C for 18–24 h. The samples were plated onto XLT4 Agar (Luqiao, Beijing, China) and incubated at 37 °C for 24 h. The plates were examined for colonies that looked like typical *Salmonella* colonies (i.e., transparent colonies with black centers). All the *Salmonella* isolates of each sample were selected and further characterized.

### 2.3. Biochemical Tests and Serotyping

An API-20E Microbial Identification Kit (bioMérieux, Shanghai, China) was used (according to the manufacturer’s instructions) to identify and differentiate members of the family Enterobacteriaceae. Isolates that exhibited typical Salmonella biochemical reactions were cultured overnight at 37 °C, and then serotyped. All the isolates were serotyped according to the White–Kauffmann–Le Minor scheme based on slide agglutination with O and H antigen-specific sera. We used diagnostic sera from a Diagnostic Sera for *Salmonella* Kit (Tianrun, Ningbo, China).

### 2.4. Antimicrobial Susceptibility Testing

The susceptibility of each of the isolates to 19 antimicrobials was assessed by determining the minimal inhibitory concentration (MIC) using the US Clinical and Laboratory Standards Institute (CLSI) broth micro dilution method. *Escherichia coli* ATCC 25922 was used as the quality control organism. The results of the tests for ampicillin (AMP), amoxicillin-clavulanate (AMC), cefazolin (CZ), kanamycin (KAN), gentamicin (GM), amikacin (AMI), tetracycline (TE), trimethoprim-sulfamethoxazole (SXT), ciprofloxacin (CIP), nalidixic acid (NAL), chloramphenicol (CHL), nitrofurantoin (NIT), meropenem (MEM) were interpreted according to CLSI M100-S25 guidelines [15], whereas those for ceftiofur (EFT), enrofloxacin (ENR), and florfenicol (FFC) were based on CLSI VET01-A4 [16]. In addition, results with the following minimum inhibitory concentration (MIC) values were considered resistant: streptomycin (STR) ≥64 µg/mL [17], olaquindox (OLA) ≥64 µg/mL [18], polymyxin B (PB) ≥2 µg/mL [19].

Intracellular MIC was determined on bacteria that were sequestered inside porcine intestinal epithelial IPEC-J2 cells. IPEC-J2 cells were plated at a density of 1 × 10^5^ cells /mL and infected with *Salmonella* at a ratio of 10 bacteria per IPEC-J2 cells. IPEC-J2 cells cultures were maintained in growth media supplemented with 100 µg/mL of gentamycin to inhibit the growth of extracellular bacteria and test antimicrobials were added to the growth media 12 h after infection. The survival of intracellular bacteria was assessed 24 h after addition of the antimicrobials. The number of surviving intracellular bacteria was determined by plating on tryptic soy agar.

All the *Salmonella* isolates were screened for PMQR genes (*qnrA, qnrB, qnrC, qnrD, qnrS, qepA, aac(6′)-Ib-cr, oqxA*, and *oqxB*), and *mcr-1* gene using a simplex PCR method. The primers used in this study are listed in Table S1. The positive products were sent for sequencing, and the DNA sequences obtained were further checked using BLAST analysis (https://blast.ncbi.nlm.nih.gov/Blast.cgi).

### 2.5. Molecular Typing

PFGE was performed in line with the US Centers for Disease Control and Prevention PulseNet protocol [20]. *Salmonella* Braenderup H9812 was used as the reference standard and *XbaI* as the restriction enzyme. The PFGE patterns were analyzed according to the Dice similarity coefficient method using BioNumerics software (version 7.6; Applied Maths, Kortrijk, Belgium). Identification of the same PFGE subtype was based on a similarity coefficient (F value) >85%. MLST was performed by using the housekeeping genes and protocol specified at the *Salmonella enterica* MLST website (http://mlst.warwick.ac.uk/mlst/dbs/Senterica).

### 2.6. Pathogenicity Analysis

Adhesion assays were adapted from Rosselin et al. [21] and modified as follows. Confluent monolayers of IPEC-J2 cells (1 × 10^5^ cells/well) were washed three times with PBS and incubated at 37 °C in 5% CO_2_ with 1 mL of *Salmonella* suspension in DMEM at 1 × 10^6^ CFU/mL to achieve a multiplicity of infection of 10:1. At 3, 6, 9 and 12 h after *Salmonella* challenge, the IPEC-J2 cells was washed twice times with PBS to remove nonadherent bacteria and then harvested by treatment with 0.05% trypsin for 10 min at 37 °C.

Internalization assays were performed as previously described [21]. Confluent monolayers of IPEC-J2 cells (1 × 10^5^ cells/well) were treated with *Salmonella* (1 × 10^6^ CFU/mL). At 3, 6, 9, and 12 h after *Salmonella* challenge, internalization was measured after an additional 2 h of incubation with DMEM supplemented with 100 µg/mL gentamicin to kill bacteria remaining outside the cells.

PCR method was used to determine the presence of 25 virulence genes (prgH, sopB, invA, sitC, spiC, sifA, misL, orfL, pipD, iroN, pefA, spvC, sipA, sipB, sipC, fliC, sopA, sipD, avrA, sptP, hilA, xthA, yafD, stn and sopE). The primers used in this study are listed in Table S2.

### 2.7. Heavy Metal Tolerance

The MIC of copper sulfate and zinc chloride was determined for selected isolates as recommended by the NCCLS guidelines. BHI agar plates containing 0, 2, 4, 8, 12, 16, 20, 24, 28, 32, 36, and 40 mM copper sulfate (CuSO_4_) adjusted to pH 7. Zinc chloride (ZnCl_2_) contained the dilution range of 0, 0.25, 0.5, 1, 2, 4, 8, and 16 mM with the pH of the medium adjusted to 5.5. Bacterial suspensions were adjusted to 10^7^ CFU/mL (100 µL of each inoculum at a 0.5 McFarland standard plus 900 µL of sterile 0.85% NaCl solutions. The plates were then incubated at 37°C for 16 to 20 h, and the growth was assessed.

PCR method was used to determine the presence of 8 heavy metal tolerance genes (*arsB, merA, pcoA, pcoD, silA, silE, tcrB*, and *terF*). The primers used in this study are listed in Table S3. The positive products were sent for sequencing, and the DNA sequences obtained were further checked using BLAST analysis (https://blast.ncbi.nlm.nih.gov/Blast.cgi).

### 2.8. Whole-Genome Sequencing and SNP Based Phylogeny

Total DNA from the *Salmonella* isolates was extracted using a TIANamp Bacteria DNA kits (Tiangen, Beijing, China) and then subjected to whole genome sequencing. The library was constructed using a Next^®^ Ultra™ DNA Library Prep kit (New England Biolabs, Ipswich, UK) according to the manufacturer’s protocol, and 250-bp paired-end reads were obtained from an Illumina Hiseq2500 platform (Bionova Biotech Co., San Diego, CA, USA). For each isolate analyzed by Whole-Genome Sequencing, at least 100-fold coverage of raw reads were collected. A draft assembly of the sequences was generated using SOAPdenovo version 2.04 (http://soap.genomics.org.cn/soapdenovo.html).

Assembled data, *S.* 4,[5],12:i:- strain SO4698-09 (RefSeq NZ_LN999997) were subjected to SNP calling and reference-based phylogeny tree building using CSI Phylogeny 1.4 [22] with SO4698-09 as the reference genome and default parameters for SNP filtering and pruning.

### 2.9. Statistical Analysis

Statistical evaluations were performed using SPSS statistical software package (version 24; IBM, Chicago, IL, USA). Chi-square test was used to compare the prevalence of *Salmonella* and antimicrobial resistance rates in various provinces. The differences among the groups in the adhesion and internalization assay were assessed using Student’s t test. For all analyses, *p*-values of less than 0.05 were considered significant.

## 3. Results

### 3.1. Prevalence and Distribution of Salmonella

**Figure 1:**
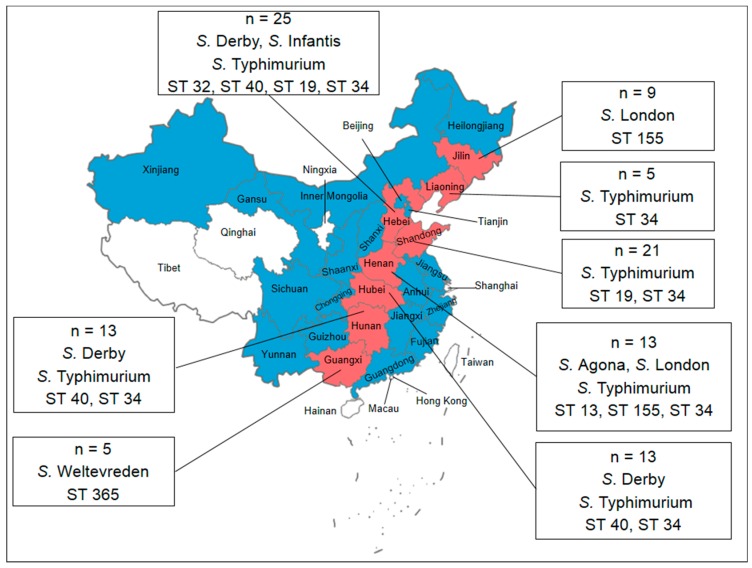
Map of China showing the samples from pigs with diarrhea from 26 provinces in China. Red in the map indicates *Salmonella*-positive sources, and the numbers indicate the numbers of *Salmonella* isolates. The data in the box indicate the distribution of the serovars and STs isolated from the pigs with diarrhea.

Between 2014 and 2016, a total of 104 *Salmonella* isolates were obtained from the intestinal contents of pigs that died of diarrhea or from the feces of pigs with diarrhea. The geographical distribution of *Salmonella* among the pigs is summarized in Figure 1. The proportion of *Salmonella* isolates was highest in Hebei (24.0%), followed by Shandong (20.2%), Hubei (12.5%), Henan (12.5%), Hunan (12.5%), Jilin (8.65%), Guangxi (4.81%) and Liaoning (4.81%). No significant difference was found in the prevalence of *Salmonella* from different provinces (*p* > 0.05).

### 3.2. Serotyping

We performed biochemical tests for each of the isolates. The serovars characterized by the biochemical tests were *Salmonella* spp. and *S. enterica* subsp. arizonae. These results were confirmed by serotyping. The H-antigens (phase 2) could not be detected in 56 (53.85%) isolates, but these isolates were identified as *S.* Typhimurium based on the MLST analysis. As a result, the 104 isolates were assigned to the following six serovars (Figure 1): *S*. Agona (2, 1.92%), *S*. Infantis (4, 3.85%), *S*. Weltevreden (5, 4.81%), *S*. Derby (8, 7.69%), *S*. London (11, 10.6%), *S*. Typhimurium (18, 17.3%) and *S.* 4,[5],12:i:- (56, 53.9%).

### 3.3. Antimicrobial Resistance

**Figure 2:**
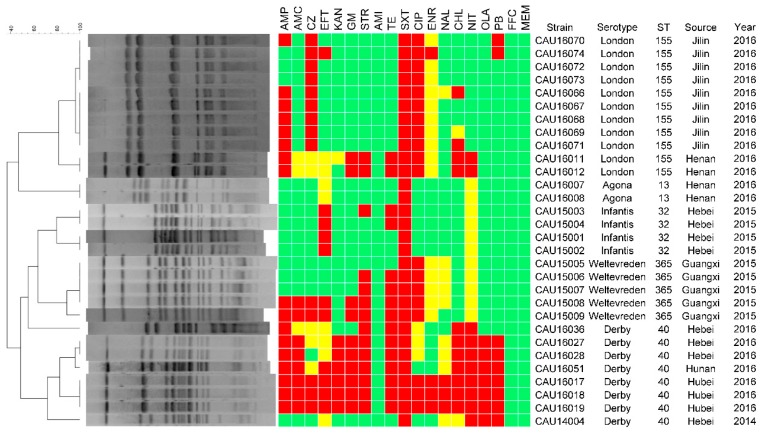
PFGE patterns and antimicrobial resistance profiles of all the *Salmonella* isolates except *S.* Typhimurium and *S.* 4,[5],12:i:-. Dendrograms of *Xba*I-PFGE are presented on the left, and the results of the antimicrobial susceptibility tests are shown to be aligned with the dendrograms. Red indicates resistance to the corresponding antimicrobials, yellow indicates intermediate susceptibility, and green indicates susceptibility.

**Figure 3:**
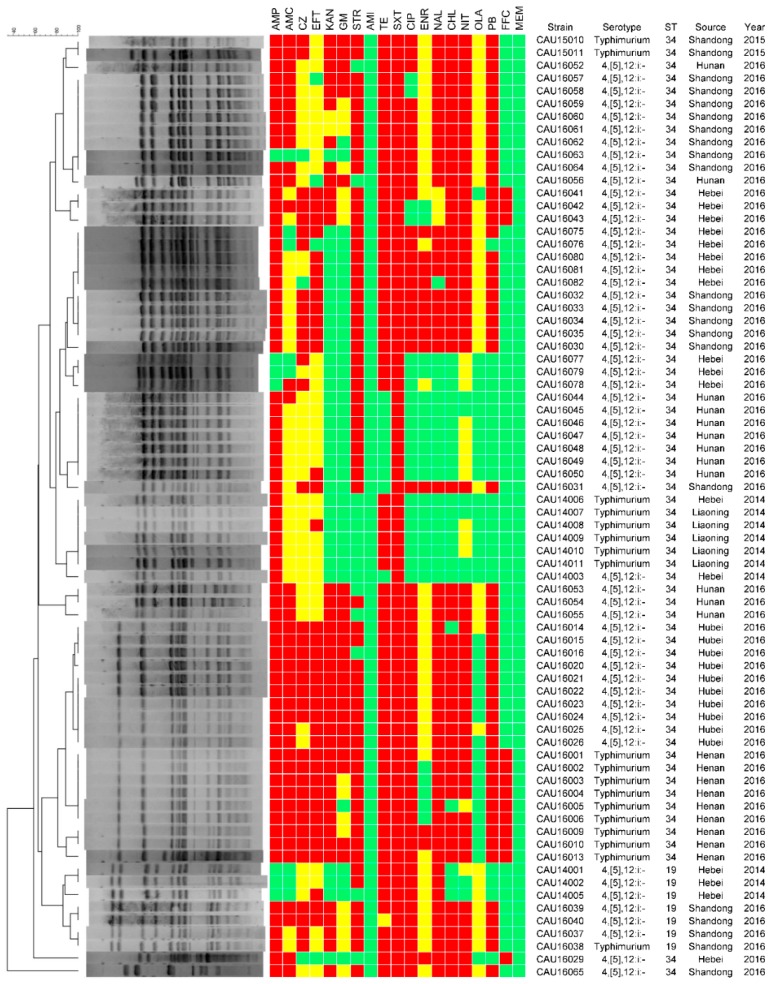
PFGE patterns and antimicrobial resistance profiles of all the *S.* Typhimurium and *S.* 4,[5],12:i:-. Dendrograms of *Xba*I-PFGE are presented on the left, and the results of the antimicrobial susceptibility tests are shown to be aligned with the dendrograms. Red indicates resistance to the corresponding antimicrobials, yellow indicates intermediate susceptibility, and green indicates susceptibility.

Antimicrobial susceptibility testing of the 104 isolates showed that they were all sensitive to amikacin and meropenem, and resistant to trimethoprim-sulfamethoxazole (Figure 2 and Figure 3). Furthermore, all the isolates showed sensitivity or intermediate resistance to nitrofurantoin. Most of the resistance among the isolates involved trimethoprim-sulfamethoxazole (n = 104, 100%), ampicillin (n = 84, 80.8%) and tetracycline (n = 80, 76.9%). They also showed resistance to the antimicrobials used only in veterinary medicine, such as florfenicol (63, 60.6%), ceftiofur (48, 46.2%), enrofloxacin (15, 14.4%).

When antimicrobial resistance was analyzed in region, the prevalence of resistance to the major tested antimicrobials among the *Salmonella* isolates were different. The prevalence of resistance to the major tested antimicrobials among the *Salmonella* isolates from Jilin and Liaoning provinces was lower than in the other provinces (*p* < 0.05). *S.* Agona from Henan province were only resistant to trimethoprim-sulfamethoxazole, intermediate susceptibility to florfenicol and ceftiofur, and sensitive to the other 16 antimicrobials.

*Salmonella* isolates resistant to at least three different classes of antimicrobials were defined as multidrug resistant (MDR) isolates. Among the 104 isolates, 73 (70.2%) showed varying degrees of MDR, and Shandong was the most MDR province (Table 1). Fortunately, *S*. Agona and *S*. Infantis do not exhibited MDR. The most common resistance profiles in *S*. Typhimurium (11, 14.9%) were AMP-TE-SXT-CHL-FFC-OLA. Among 18 *S*. Typhimurium isolates, 12 isolates with the ASSuT genotype. Among 56 *S.* 4,[5],12:i:- isolates, the ASSuT genotype was observed in 34 isolates.

Among 104 *Salmonella* isolates, *qnrB*, *qnrS*, *aac(6′)-Ib-cr*, *oqxA* and *oqxB* were observed, *aac(6′)-Ib-cr* was the dominant PMQR gene (84, 80.8%), followed by *qnrS* (49, 47.1%), *qnrB* (32, 30.8%), *oqxA* (22, 21.2%) and *oqxB* (22, 21.2%). Phenotypic resistance to nalidixic acid, ciprofloxacin and enrofloxacin was found with the presence of *qnrB, qnrS* and *aac(6′)-Ib-cr.* The *oqxAB* gene was observed in *S.* 4,[5],12:i:-, and *S*. Typhimurium without *oqxAB* gene. All *Salmonella* isolates were tested for the presence of *mcr-1* gene, *mcr-1* gene was observed in 12 isolates. Isolates of *Salmonella* strains that carried *mcr-1* genes only showed *S.* Typhimurium and *S.* 4,[5],12:i:-. All *mcr-1*-positive isolates were colistin-resistant with MIC of 8–16 µg/mL.

**Figure 4:**
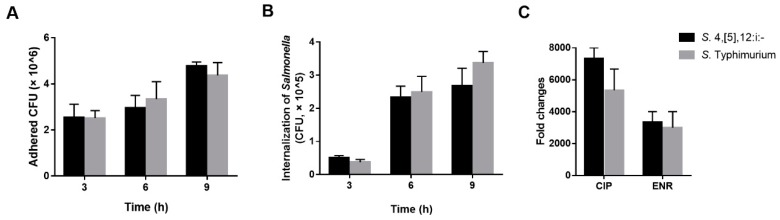
Adhesion to and internalization into IPEC-J2 of *S.* Typhimurium and *S.* 4,[5],12:i:-. *Salmonella* populations adhered to (**A**) and internalized into (**B**) IPEC-J2 were determined after 3, 6 and 9 h of interaction, respectively. The fold change of intracellular MIC to extracellular MIC for *S.* Typhimurium and *S.* 4,[5],12:i:- (**C**). Each experiment was done in triplicate, and differences between groups were compared using Student’s t test.

Similar antimicrobial resistance patters were observed in *S.* Typhimurium and *S.* 4,[5],12:i:-. Of 18 *S.* Typhimurium isolates, high antimicrobial resistance rate were found for ampicillin, tetracycline, and trimethoprim-sulfamethoxazole; and 18 of 18 *S.* Typhimurium were MDR isolates. The *aac(6′)-Ib-cr* gene was the dominant PMQR genes (12, 66.7%), followed by *qnrS* (10, 55.6%). Among 56 *S.* 4,[5],12:i:- isolates, high antimicrobial resistance rate was found for tetracycline, trimethoprim-sulfamethoxazole, and florfenicol; and 56 out of 56 were MDR isolates. The *aac(6′)-Ib-cr* gene was the dominant PMQR genes (32, 57.1%), followed by *qnrS* (25, 44.6%). No significant difference (*p* > 0.05) has been showed in the presence of PMQR genes between *S.* Typhimurium and *S.* 4,[5],12:i:-. We determined the ciprofloxacin and enrofloxacin MIC for intracellular *S*. Typhimurium and *S.* 4,[5],12:i:- (Figure 4). We found that the ciprofloxacin and enrofloxacin MIC for intracellular *Salmonella* was significantly higher than extracellular *Salmonella*. However, no significant difference (*p* > 0.05) has been showed in the intracellular MIC between *S*. Typhimurium and *S.* 4,[5],12:i:-.

### 3.4. Molecular Typing

For the PFGE analysis, the 104 isolates were divided into two groups. The PFGE typing of the first 30 isolates using *Xba*I led to their categorization into three clusters and ten PFGE subtypes (Figure 2). Strains from the same farm that belonged to the same serovar were highly genetically related. However, the similarity coefficient of strains from the same farm that belonged to different serovars was low. The PFGE typing of the 74 *S*. Typhimurium and *S.* 4,[5],12:i:- isolates by *Xba*I led to their categorization into seven clusters (Figure 3). The resultant dendrogram of *Salmonella* isolates exhibited clusters with a high level of diversity. A part of the similarity coefficient of *Salmonella* isolated from the same farms was lower. The similarity coefficients between the *Salmonella* isolates were relatively low whether or not the isolates were from the same province or the same year. However, we also found that the similarity coefficient for the *Salmonella* isolates from different provinces was >90%. Whether or not the isolates were from the same province, the *S*. Typhimurium isolates were highly similar genetically to *S.* 4,[5],12:i:-.

Based on the *Salmonella* MLST scheme associated with the University of Warwick MLST database, seven STs were identified among the 104 *Salmonella* isolates investigated, namely, ST34, ST32, ST19, ST155, ST40, ST13, and ST365 (Figure 4). The majority of isolates (67 isolates, 64.4%) were assigned to ST34. Eleven isolates (10.6%) belonged to ST155, eight (7.69%) to ST40, seven (6.73%) to ST19, five (4.81%) to ST365, four (3.85%) to ST32, and two (1.92%) to ST13. One *S*. Typhimurium and six *S.* 4,[5],12:i:- isolates belonged to ST19.

### 3.5. Pathogenicity Analysis

We tested in vitro the adhesion and internalization abilities of *S*. Typhimurium and *S.* 4,[5],12:i:- (Figure 4). Several time points of adhesion and internalization were tested to evaluate the capacities of *Salmonella* adhesion to IPEC-J2 cells and internalization into IPEC-J2 cells. At 3 h after *Salmonella* challenge, we found that there is no significant difference (*p* > 0.05) in the capacities of adhesion and internalization between *S*. Typhimurium and *S.* 4,[5],12:i:-. As a comparison, adhesion and internalization capacities of 6 h, 9 h, and 12 h were also assessed. The adhesion and internalization capacities of *S*. Typhimurium were the same as *S.* 4,[5],12:i:- at different time points.

All the *S*. Typhimurium were positive for 24 virulence genes tested. Among 56 *S.* 4,[5],12:i:- isolates, the *pefA* gene was the least prevalent of all the virulence genes (85.7%), followed by *spvC, invA, avrA, sipA, fliC, misL* and *yafD*. The *sopE* gene was observed only in *S.* 4,[5],12:i:- isolates (14, 25.0%).

### 3.6. Heavy Metal Tolerance

Among 74 *Salmonella* isolates, *pcoA, pcoD, silA, silE* and *terF* were observed in all isolates. The merA gene was only found in *S.* 4,[5],12:i:- isolates. No significant difference in the MIC for copper sulfate and zinc chloride between *S*. Typhimurium and *S.* 4,[5],12:i:- could be seen.

### 3.7. Phylogenetic Analysis

**Figure 5:**
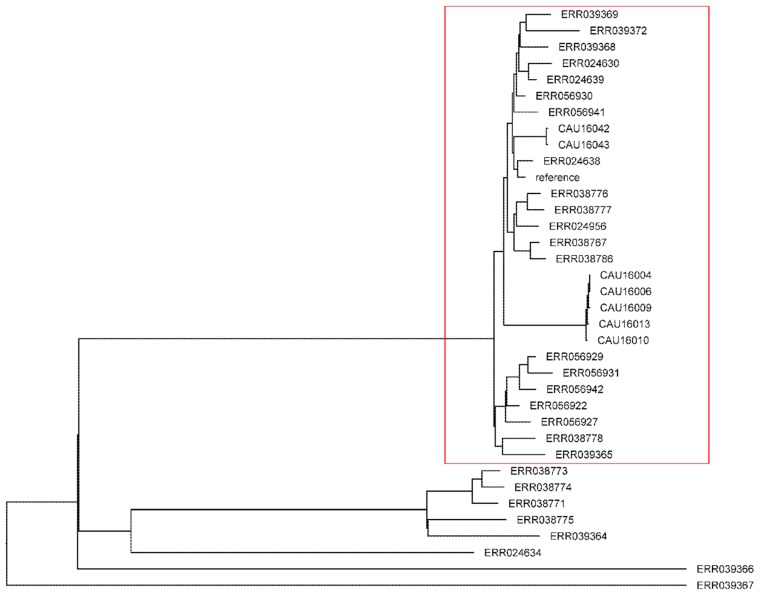
Phylogenetic tree obtained from SNP based phylogeny when SO4698-09 is used as reference strain. Red: Cluster 1.

Single Nucleotide polymorphism (SNP) based on phylogeny using SO4698-09 as a reference genome, which is a representative multidrug European clone revealed 2211 high-quality shared SNP positions between each strain and this reference genome [13]. A maximum likelihood tree was constructed using *S*. Typhimurium, *S.* 4,[5],12:i:- and the 28 *S.* 4,[5],12:i:- from Europe. As shown in Figure 5, one main clusters were identified. Cluster 1 comprises SO4698-09 and 27 strains, none of which was ST19; this cluster included 2 *S.* 4,[5],12:i:-, 5 *S*. Typhimurium isolates, and 20 *S.* 4,[5],12:i:- from Europe. *S.* 4,[5],12:i:- and *S*. Typhimurium isolates were interspersed with *S.* 4,[5],12:i:- isolates of Europe origin.

## 4. Discussion

In this study we identified 104 *Salmonella* isolates from pigs in 66 farms in 8 provinces of China (out of the 26 provinces investigated). In order of frequency, these isolates belonged to *S.* 4,[5],12:i:-, *S*. Typhimurium, *S*. London, *S*. Derby, *S*. Weltevreden, *S*. Infantis and *S*. Agona. Almost all the *Salmonella* isolates were resistant to a large majority antimicrobials. The PFGE and MLST results indicated that the *Salmonella* isolates from different regions were genetically diverse. *S.* 4,[5],12:i:- currently circulating in pig in China are most likely part of a European clade, which are mainly ST34 and harbors ASSuT genotype, heavy metal tolerance genes, and, to an extent, PMQR genes, *mcr-1* gene, *sopE* gene.

The *Salmonella* isolation rate for pigs assessed in this study is higher than in previous studies in Shandong province (7.23%) [23], in central China (4.09%) [24], in Sichuan province (16.4%) [25], in the European Union (EU) (7.44%) [26], and in the USA (8.79%) [27]. The differences in these isolation rates might be caused by a variety of factors. Sample types (e.g., feces and intestinal contents), collection seasons and isolation methodologies can limit the comparison of different samples of *Salmonella* isolates. Therefore, it is essential to establish a global standard protocol for the isolation of *Salmonella* strains, especially for long-term monitoring of *Salmonella* infections.

The *Salmonella* serovars differed by region. In this study, the most commonly isolated serovar in pigs with diarrhea was *S.* 4,[5],12:i:-. In contrast, in previous studies, the dominant serovar in pigs was *S.* Derby in Shandong province [23]; IIIb in central China [24]; *S.* Adelaide in the USA [27]; and *S.* Typhimurium in the EU [26]. The differences in the dominant serovars in pigs may be due to differences in geographical regions. According to the WHO Global Foodborne Infections Network (GFN) Country Databank (http://www.who.int/gfn/activities/en/), the dominant serovar in foodstuffs in China was *S.* Derby. However, the main serovar in foodstuffs in the USA was *S.* Typhimurium, and it was *S.* Infantis in Japan. Thus, it is clear that each country or region can have different serovars, and different diversities and complexities.

Antimicrobial resistance is one of the biggest threats to global health, food security, and development. The extensive use and misuse of antimicrobials in animals exert a selection pressure on bacteria such as *Salmonella*, so the antimicrobial-resistant strains are more likely to survive. Consistent with other studies carried out in China [23,24,25], the *Salmonella* isolates were most likely to be resistant to trimethoprim-sulfamethoxazole and less likely (but still likely) to be resistant, to ampicillin, tetracycline, nalidixic acid, chloramphenicol and florfenicol. The high frequency of resistance to trimethoprim-sulfamethoxazole, tetracycline, and florfenicol may be related to the long-term use of these antimicrobials in China, and the incorporation of antimicrobials into animal feed so that they are present in animals at therapeutic levels in order to prevent bacterial disease.

In agreement with the findings of a previous study in Sichuan province, *S*. Typhimurium were more likely to be resistant to four antimicrobials or more [25]. When resistance profiles and serovars or geographical regions were compared, we found that there appeared to be a relationship between antimicrobial resistance and particular serovars or regions.

In general, the frequency of resistance of the *Salmonella* isolates to each of the 19 tested antimicrobials was lower in Jilin and Liaoning provinces than in the other provinces. The isolates in these two provinces were sensitive to all the antimicrobials tested except ampicillin, cefazolin, tetracycline, trimethoprim-sulfamethoxazole and ciprofloxacin. The frequencies of antimicrobial resistance in Guangxi province were similar to in Jilin and Liaoning provinces. However, in contrast to the other *Salmonella* isolates from Guangxi province, there were two isolates from this province that were resistant to ampicillin, amoxicillin-clavulanate, cefazolin, ceftiofur and gentamicin. This may be partly due to the use of different kinds and doses of antimicrobials for the treatment and prevention of diseases in pigs at different farms.

The antimicrobial resistance results for Shandong province and central China (Henan, Hubei, and Hunan provinces) were similar to those found in earlier studies in Shandong province [23] and Henan, Hubei, and Hunan provinces [24]. This similarity may be caused by the relatively similar usage in these areas (over time) of antimicrobials for disease control and prevention during the pig breeding process. Another potential factor is that the number of live pigs is significantly higher in central China than in other areas of China [28]. As China is one of the major pig breeding countries, the demand for and use of antimicrobials are huge. Therefore, there is a higher risk of antimicrobial misuse. Measures should be taken to further regulate the use of antimicrobials in order to guarantee food safety and public health.

Consistent with the finding in previous studies, the *mcr-1*-positive *Salmonella* isolates were MDR strains, and it carry fluoroquinolones resistance genes [29]. The *mcr-1*-positive *Salmonella* isolates from Henan and Hebei province carried *aac(6′)-Ib-cr* and *oqxAB*, respectively. All the *mcr-1*-positive isolates were resistant to more antimicrobials than the *mcr-1*-negative isolates were. Studies have shown that there is a correlation between the rises in antimicrobial resistance among pathogenic bacteria and the use of antimicrobials in animals feeds [30]. To improve health and promote growth, colistin (together with other antimicrobials) was frequently used in feed. This increasingly heavy use of antimicrobials could have resulted in high selective pressure in environment and led to the emergence of MDR.

From January 1, 2006, the EU withdrew its approval for the use of antimicrobial growth promoters in animal feed. In 2012, the US Food and Drug Administration (FDA) banned the use of the cephalosporin class of antimicrobials in food-producing animals. Likewise, China has also taken measures to prevent the emergence of drug-resistant bacteria and promote food safety and public health. For instance, China issued an order prohibiting certain use of four fluoroquinolones (lomefloxacin, ofloxacin, norfloxacin and pefloxacin) in food-producing animals in 2015 and the use of colistin as a growth promoter in 2016. However, it is necessary to develop more strategies and draft more regulations to control the use of antimicrobials and prevent the emergence of drug-resistant bacteria.

In this study, in addition to *S.* Typhimurium 4,[5],12:i:1,2, a monophasic variant known as serovar 4,[5],12:i:- was detected, and this monophasic variant was more dominant than *S.* Typhimurium. The frequency of antimicrobial resistance genotypes was high among *S.* 4,[5],12:i:- isolates, and with the presence of an ASSuT genotype, typical of the European clone.

The *sopE* gene was found in 14 of 56 of *S.* 4,[5],12:i:-. The result was in agreement with the previous studies [13]. The acquisition of the *sopE* gene may affect the pathogenicity, but there is no significant difference between *S.* Typhimurium and *S.* 4,[5],12:i:- in adhesion and internalization assays. The expression of *sopE* may increase the fitness of *Salmonella*, and the transmission of *Salmonella* by induce inflammatory diarrhea. Furthermore, *sopE* expression results in increased amounts of salmonellae in the intestinal lumen and shedding in the feces, and increased production of host nitrate.

Not only encoded virtually *S.* Typhimurium heavy metal tolerance genes but also *S.* 4,[5],12:i:- did. Assembled data were searched for a genomic island of resistance to heavy metals (SGI-3) and all 7 strains encoded SGI-3. Supplements such as copper and zinc, are added in pig feed as micronutrients and general antimicrobials to increase feed efficiency and to promote animal growth. In the EU, heavy metals have been used increasingly since the ban of the nonspecific use of antimicrobials as growth promoters in pig feed [31]. Heavy metals are very persistent in the environment [32], and concentration of heavy metals in pig intestines may represent substantial selective pressure contributing to the success of *Salmonella*.

The MLST analysis identified two *S.* Typhimurium clonal groups from different sources that differed only in terms of one of the profiled housekeeping genes. Furthermore, *S.* Typhimurium 4,[5],12:i:1,2 and 4,[5],12:i:- did not correspond to only one ST. The strains of ST34 were clustered into a single pattern. Therefore, we hypothesized that these strains persisted in specific regions and continually proliferated within these regions.

Currently, *S.* Typhimurium 1,4,[5],12:i:- is one of the most common *Salmonella* serovars isolated from humans and animals in many countries. For example, it was the third most common serovar responsible for human and animal infections in the EU [26] and the fifth most common in USA [27]. In China, since 2009, *S.* 4,[5],12:i:- has been one of the four most common serovars to cause human salmonellosis [33]. Pigs are considered one of the main vectors in the spread of *S.* Typhimurium 1,4,[5],12:i:- [34]. In order to better control salmonellosis, we should monitor the emergence, frequency, and variation of the various *Salmonella* serovars.

## 5. Conclusions

The present study provides significant information regarding the prevalence and characteristics of *Salmonella* isolated from pigs with diarrhea in China for the first time. Co-occurrence of PMQR and *mcr-1* encoding genes in *Salmonella* isolates were identified. The level of antimicrobial resistance observed in this study further highlights the need for careful and reasonable use of antimicrobials. The high frequency and rapid increase of *S.* 4,[5],12:i:- may represent a public health concern.

## Figures and Tables

**Table 1:** The number of multidrug-resistance *Salmonella* isolates from different provinces unit: Strain.

Resistance Pattern	Guangxi	Liaoning	Jilin	Henan	Hubei	Hunan	Shandong	Hebei
3	0	5	2	0	0	0	0	1
4	0	0	0	2	0	0	1	2
5	2	0	0	0	3	6	11	4
6	0	0	0	1	6	0	3	2
7	0	0	0	8	1	0	6	4
8	0	0	0	0	3	0	0	0
Total	2	5	2	11	13	6	21	13

## Supplementary Materials

The following are available online at http://www.mdpi.com/2076-2607/6/4/117/s1, Table S1: Primers uesd for the amplification of the antimicrobial resistance genes, Table S2: Primers uesd for the amplification of the *Salmonella* virulence genes, Table S3: Primers uesd for the amplification of the heavy metal tolerance genes.
