# Supplementary material for: Distribution and Antimicrobial Resistance of Salmonella Isolated from Pigs with Diarrhea in China
Source: Microorganisms. 2018 Nov 26;6(4):117. doi: 10.3390/microorganisms6040117 (PMC6313467; doi:10.3390/microorganisms6040117)
Supplement: Supplementary file 1 [file microorganisms-06-00117-s001.pdf]

Table S1. Primers uesd for the amplification of the antimicrobial resistance genes.

Gene	Direction	Primer sequences (5' to 3')	Product length (bp)
qnrA	Forward	TTCAGCAAGAGGATTTCTCA	628
	Reverse	GGCAGCACTATTACTCCCAA	
qnrB	Forward	CCTGAGCGGCACTGAATTTAT	409
	Reverse	GTTTGCTGCTCGCCAGTCGA	
qnrC	Forward	GGGTTGTACATTTATTGAATC	447
	Reverse	TCCACTTTACGAGGTTCT	
qnrD	Forward	TTACGGGGAATAGAGTTA	468
	Reverse	AATCAGCCAAAGACCAAT	
qnrS	Forward	GCAAGTTCATTGAACAGGGT	428
	Reverse	TCTAAACCGTCGAGTTCGGCG	
aac(6')-Ib-cr	Forward	TTGCGATGCTCTATGAGTGGCTA	482
	Reverse	CTCGAATGCCTGGCGTGTTT	
qepA	Forward	CCAGCTCGGCAACTTGATAC	570
	Reverse	ATGCTCGCCTTCCAGAAAA	
oqxA	Forward	GATCAGTCAGTGGGATAGTTT	670
	Reverse	TACTCGGCGTTAACTGATTA	
oqxB	Forward	TTCTCCCCCGGCGGGAAGTAC	512
	Reverse	CTCGGCCATTTTGGCGCGTA	
mcr-1	Forward	ATCAGCCAAACCTATCCTATCG	1257
	Reverse	ATAGATGTTGCTGTGCGTCTGC	

Table S2. Primers uesd for the amplification of the *Salmonella* virulence genes.

Gene	Direction	Primer sequences (5' to 3')	Product length (bp)
prgH	Forward	GCCCGAGCAGCCTGAGAAGTTAGAAA	756
	Reverse	TGAAATGAGCGCCCCTTGAGCCAGTC	
sopB	Forward	GAAGACTACCAGGCGCACTT	805
	Reverse	TTGTGGATGTCCACGGTGAG	
invA	Forward	CTGGCGGTGGGTTTTGTTGTCTTCTCTATT	1070
	Reverse	AGTTTCTCCCCCTCTTCATGCGTTACCC	
sitC	Forward	CAGTATATGCTCAACGCGATGTGGGTCTCC	768
	Reverse	CGGGGCGAAAATAAAGGCTGTGATGAAC	
spiC	Forward	CCTGGATAATGACTATTGAT	301
	Reverse	AGTTTATGGTGATTGCGTAT	
sifA	Forward	TTTGCCGAACGCGCCCCCACACG	449
	Reverse	GTTGCCTTTTCTTGCGCTTTCCACCCATCT	
misL	Forward	GTCGGCGAATGCCGCGAATA	561
	Reverse	GCGCTGTTAACGCTAATAGT	
orfL	Forward	GGAGTATCGATAAAGATGTT	332
	Reverse	GCGCGTAACGTCAGAATCAA	
pipD	Forward	CGGCGATTCATGACTTTGAT	399
	Reverse	CGTTATCATTCGGATCGTAA	
iroN	Forward	ACTGGCACGGCTCGCTGTCGCTCTAT	1205
	Reverse	CGCTTTACCGCCGTTCTGCCACTGC	
pefA	Forward	GCGCCGCTCAGCCGAACCAG	156
	Reverse	CAGCAGAAGCCCAGGAAACAGTG	
spvC	Forward	TCTCTGCATTTCGCCACCAT	563
	Reverse	TGCACAACCAAATGCGGAAG	
sipA	Forward	TACCCCTGCTGCTACGTAAT	917
	Reverse	CTCCAGGGCTTTACGTATCA	
sipB	Forward	TGGCAGGCGATGATTGAGTC	614
	Reverse	CCCATAATGCGGTTCGTTTC	
sipC	Forward	TGCCCTGGCAAATAATGTCA	689
	Reverse	CATCGATTCGGGTCATATCC	
fliC	Forward	TACGCTGAATGTGCAACAAA	554
	Reverse	TACCGTCATCTGCAGTGTAT	
sopA	Forward	GCCCACGGTTTCTGAAGGTA	982
	Reverse	AAAGAGTCCGCTGTGAGTGG	
sipD	Forward	TGCGTCAGCGTCTGTAATGT	588
	Reverse	GGCCTTATTTAGCGCTTCGC	
avrA	Forward	ATACTGCTTCCCGCCGC	667
	Reverse	ACACCGAAGCATTGACCTGT	
sptP	Forward	TTCACCCTATCCGCCAGGTA	658
	Reverse	GTGTAGCCCGGTTCTCACAA	
hilA	Forward	CACCAACCCGCTTCTCTCTT	345
	Reverse	ATTGTGGTCCAGCTCTGTCG	
xthA	Forward	CGAAAAACACCAGCCCGATG	479

	Reverse	CCGGCAGGAAGGAGCATTTA	
yafD	Forward	CGGATCCGTATCCTCGTGTG	531
	Reverse	ATCGTCAGTGAAACGCACCT	
stn	Forward	CTTTGGTCGTAAAATAAGGCG	260
	Reverse	TGCCCAAAGCAGAGAGATTC	
sopE	Forward	CCAAGAATGACGTTTTTAGCCCA	199
	Reverse	TGCTGTGGAGTCGGCATAG	

Table S3. Primers uesd for the amplification of the heavy metal tolerance genes.

Gene	Direction	Primer sequences (5' to 3')	Product length (bp)
arsB	Forward	GCACGGTCGGCATATTGTTC	549
	Reverse	TGGTGAATATCGTCTCGGCG	
merA	Forward	AGCGAGACGATTCCTAAGCG	849
	Reverse	GAGCTTCAACCCTTCGACCA	
pcoA	Forward	TGGTGAAACGGCCGTCAATA	565
	Reverse	CGCGAGATCAGTCGGATTCA	
pcoD	Forward	GCTGGATTGTCAGGCTCTGT	514
	Reverse	ACCATAACTTCAAGCCGGGG	
silA	Forward	AAAAACGCGCGTGACGTAAT	618
	Reverse	CAGGATCGGAATGACAATGATGG	
silE	Forward	GGGCCAGACTGACCGTTATT	529
	Reverse	CAATATCCGGCGTCAGTGGA	
tcrB	Forward	CACCACCATCACGGTAGCTT	989
	Reverse	GCGTCCCGGTCTTTCACTAA	
terF	Forward	AGCATGCAGGCTCAAGGAAT	582
	Reverse	CTCGTTGTTTGTCCCTCCGA	
